# Loss of Thy1 in Cortico-Striatal Pathways Alters Response to Dopamine and Gabapentin

**DOI:** 10.1080/17590914.2026.2615452

**Published:** 2026-01-15

**Authors:** Cezar Goletiani, Matthew D. McEchron, Elizabeth Neely, James R. Connor

**Affiliations:** a Institute of Cognitive Neurosciences, Free University of Tbilisi, Tbilisi, Georgia; b V. Bakhutashvili Institute of Medical Biotechnology, Tbilisi State Medical University, Tbilisi, Georgia; c Department of Neurosurgery, Pennsylvania State University, College of Medicine, Hershey, PA, USA; d Department of Preclinical Curriculum and Assessment, College of Osteopathic Medicine, Rocky Vista University, Englewood, CO, USA

**Keywords:** Dopaminergic modulation, RLS, startle reactions, striatum, synaptic efficacy, Thy1 KO mouse

## Abstract

Thy1, a synaptic protein, may support synaptic junction adherence. Thus, we hypothesized that loss of Thy1 may alter synaptic transmission. Our focus on the Thy1 knockout (KO) mouse model stems from the loss of Thy1 expression in individuals with Restless Legs Syndrome (RLS), a neurological disorder. This investigation aimed to determine: 1) if the absence of Thy1 affects synaptic function in the striatal region, 2) if the absence of Thy1 alters the synaptic response to dopamine and gabapentin, and 3) if the Thy1 loss can alter behavior modulated by the striatum. Network-level synaptic transmission was measured in corticostriatal slices from Thy1 KO and C57BL/6 control mice. *In vivo*, acoustic startle behavioral testing was used to measure startle reaction and prepulse inhibition in both groups. Raclopride, a D_2_ receptor antagonist, decreased population spike amplitude in control but not Thy1 KO slices. Quinpirole, a D_2_ receptor agonist, did not change spike amplitude in any group. Gabapentin, a Ca^2+^ channel blocker, reduced population spike amplitude in Thy1 KO slices more than in controls. The behavioral acoustic startle response was diminished in Thy1 KO mice and attributed to enhanced prepulse inhibition. Loss of Thy1 alters striatal synaptic function, affecting dopaminergic modulation of corticostriatal neurotransmission and resulting in disruption of the startle response and prepulse inhibition.

## Highlights

- Loss of Thy1 affects dopaminergic modulation of corticostriatal neurotransmission.
- Loss of Thy1 alters the pharmacological response to dopaminergic agents and gabapentin.
- Loss of Thy1 affects startle response to auditory stimuli.
- Thy1 knockout in mice affects neural mechanisms common to restless legs syndrome.

## Introduction

Thy1 is a glycosyl-phosphatidylinositol (GPI)-linked integral membrane protein of the immunoglobulin superfamily and is expressed by lymphocytes, endothelial cells, fibroblasts, and many cancer cells (Rege & Hagood, 2006a, 2006b). It is also expressed at high levels (estimated at 7.5% of neuronal membrane protein) in nervous system tissue in virtually all mammalian species (Morris, 1985; Morris et al., 1983) and is enriched in synaptosomal fractions of the brain (Stohl & Gonatas, 1977). Thy1 expression precedes maturation of dopaminergic terminals and may serve as a guide for neuronal outgrowth in the striatum as part of its role as an anchor protein in lipid raft membrane structures (Mann et al., 1989; Shults & Kimber, 1993).

The motivation for this study was the observation that Thy1 is decreased in specific brain regions obtained from autopsy samples of humans diagnosed with Restless Legs Syndrome (RLS) (Barrière et al., 2005; Wang et al., 2004). RLS is a chronic sensorimotor neurological disorder characterized by uncomfortable leg sensations followed by an urge to move the legs. The precise etiology of RLS is unclear, but clinical and autopsy evidence suggests that dopaminergic dysfunction in the striatum may play a role in RLS symptomatology (Connor et al., 2009; Earley et al., 2013; Michaud et al., 2002). There is an altered dopaminergic profile in the striatum in the thy1 KO mouse model (Connor et al., 2008). Although Thy1 has not emerged as a major primary genetic susceptibility factor for RLS in genome-wide association studies — unlike MEIS1, BTBD9, and MAP2K5/SKOR1 (German Mouse Clinic Consortium, 2017; Li et al., 2017; Trenkwalder et al., 2018) — the current study uses Thy1 KO mice specifically as a model of Thy1 deficiency rather than as a disease model of RLS. Our goal was to determine whether the absence of Thy1 expression alters pharmacologically specific synaptic activity in the striatum and striatal modulation of sensorimotor gating, and to assess whether any such changes intersect with pathways implicated in RLS, without implying etiological equivalence (Connor et al., 2009; Ward et al., 1995). Network-level synaptic efficacy was measured electrophysiologically from the striatum of Thy1 KO and control mice using the corticostriatal slice technique (Calabresi et al., 1997; Hawes et al., 2013). This technique enables precise control of the medium that bathes the slice, allowing for manipulation of the pharmacological milieu that dictates synaptic function.

Two of the major targeting systems influencing striatal functions may be the dopaminergic or GABAergic systems (Mercuri et al., 1985; Trenkwalder, 2006; Winkelman, 2006). Thus, D_2_-receptor function was examined in the corticostriatal slice using D_2_-specific agonists and antagonists. We also examined synaptic responses to gabapentin, the drug commonly used to treat several neurological disorders, which blocks voltage-dependent Ca^2+^ channels (VDCCs) resulting in indirect modulation of striatal inhibitory network activity (Cheng & Chiou, 2006; Happe et al., 2003; Hundehege et al., 2018). Input-output (IO) curves were used to examine the effect of each pharmacological agent in the corticostriatal slice. IO curves standardized the stimulation input current from the synaptic threshold to the maximum population spike amplitude. This allowed for an objective comparison of synaptic function between groups. We also examined *in vivo* striatal function in the Thy1 KO mouse using acoustic startle response (ASR) and prepulse inhibition (PPI) behavioral tests, which are considered to be modulated by the striatum (De Haan et al., 2018; Frauscher et al., 2007).

## Materials and Methods

### Generation of Transgenic Mice

Thy1 null mutant mice were obtained as a generous gift from Dr. Kyoko Hayakawa (Fox Chase Cancer Center, Philadelphia, PA). To generate the mice, Thy1 was inactivated in C57BL/6 ES cells using a targeting vector and strategy already described for 129/Sv/Ev ES cells (Nosten-Bertrand et al., 1996). In our laboratory, the mice were bred and genotyped by Southern blotting (Nosten-Bertrand et al., 1996). Wild-type C57BL/6 mice were used as control animals, as this strain represents the genetic background of the Thy1 knockout mice (Kollias et al., 1987).

### Brain Slice Preparation

Synaptic efficacy was examined in corticostriatal slices of male Thy1 KO and background control (C57BL/6) mice (aged 6–18 mo). One control and one experimental mouse were used for each day of recording. Slices were obtained from a minimum of 5 mice from each experimental group. All experiments were carried out according to the Pennsylvania State University Animal Care and Use Committee and were in accordance with the guidelines specified by the National Institute of Health Guide for the care and use of laboratory animals.

Preparation and maintenance of brain slices have been described previously (Calabresi et al., 1997; Hawes et al., 2013). Mice were deeply anesthetized with 4.0% gaseous halothane in 100% O_2_ and decapitated. The brain was (in <25 s) removed rapidly and placed in pre-aerated (95% O_2_ and 5% CO_2_) ice-cold dissecting solution. The composition of dissecting solution is as follows (all values in mM): 234 Sucrose, 3.6 KCl, 1.2 MgCl_2_, 2.5 CaCl_2_, 1.2 NaH_2_PO_4_, 25 NaHCO_3_ and 12 glucose (pH 7.4). Coronal corticostriatal slices (500 µm) were cut with a vibratome and incubated in a holding chamber with aerated artificial cerebrospinal fluid (ACSF) for 1 h at 32 °C. The composition of the ACSF was as follows (all values in mM): 125 NaCl, 2.5 KCl, 1 MgCl_2_, 2 CaCl_2_, 1.25 NaH_2_PO_4_, 26 NaHCO_3_, 25 glucose (pH 7.4). During electrophysiological recording, individual slices were transferred into a recording chamber, completely submerged in circulating ACSF.

### Pharmacological Agents

All pharmacological agents were premixed with ACSF prior to recording. All of the drugs were purchased from Sigma (St. Louis, MO). The role of the D_2_ dopaminergic receptor was investigated using the D_2_ agonist quinpirole (0.5, 3, and 15 μM) or the antagonist raclopride (5 μM). Voltage-dependent Ca^2+^ channels were blocked with 300 or 600 μM gabapentin. The concentration of all drugs was adopted from other corticostriatal recording studies (Bamford et al., 2004; Garcia-Munoz et al., 1991a, 1991b; Gardoni & Bellone, 2015).

### Corticostriatal Population Spikes

**Figure 1.**
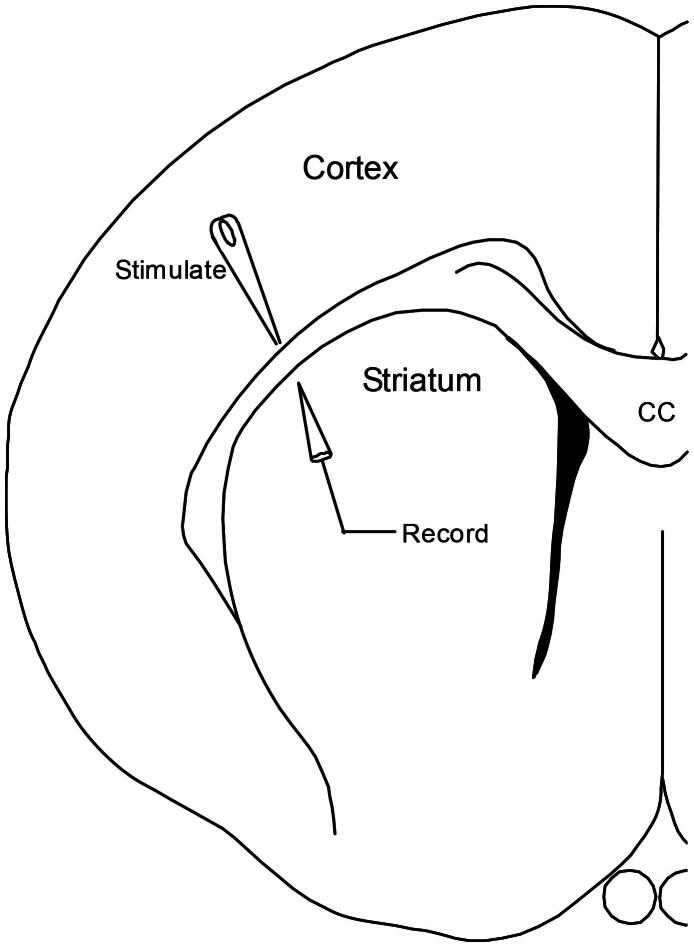
The diagram shows the approximate placement of stimulating and recording electrodes used to evoke population spikes in the corticostriatal slice.

During electrophysiological recording, corticostriatal slices were submerged in a recording chamber and continuously perfused at 5 ml/min with warmed (35 °C) aerated ACSF. A computer-controlled analog stimulus isolation unit (A-M Systems 2200; Carlsborg, WA) was used to deliver constant current bipolar pulses (0.1 ms/phase) through two Teflon insulated tungsten wires (75 μm diameter each) bonded together. Figure 1 shows electrode arrangement for recording corticostriatal population spikes. Stimulation was delivered to the lateral section of corpus callosum. Population spikes were recorded extracellularly from the lateral striatum using a glass pipette (tip diameter 20 μm) filled with 0.9% NaCl. Population spikes were amplified (1000X; A-M Systems Model 1800), filtered between 10 Hz and 1 kHz, and sampled by a DT3010 data acquisition board (Data Translation; Marlboro, MA) at 20 kHz.

The amplitude of the population spikes was defined as the difference between the peak negative voltage and the subsequent positive peak voltage. The procedures for measuring the population spike amplitude were based on methods described previously (Breton et al., 2015; Horne et al., 1990). IO curves were measured by delivering a range of current intensities between 10 and 600 μA in 20 μA steps (30 s between). Data were collected from population spikes that had an amplitude greater than 500 μV and had a latency between 4 and 9 ms. Slices were allowed to stabilize for 10 min before data were collected. Stable population spikes were subjected to a full baseline IO curve, followed 10 min later by a full IO curve with a pharmacological agent in the bath.

### Startle Behavior

ASR, PPI and latency of maximal response (Tmax) were examined in Thy1 KO mice and in the background strain C57BL/6. Mice were housed in single cages in isolation chambers in rooms in which there was control over light-dark cycles, temperature and humidity. Light-dark cycles were 12 h/12 h. Mice had unlimited access to food and water *ad libitum*.

Experiments were carried out on a special experimental startle platform. The startle boxes (SR-Lab, San Diego Instruments, San Diego, CA) consisted of nonrestrictive plexiglas cylinders resting on a platform inside of a ventilated chamber. Mice were first acclimated to the background noise (white noise) of 70 dB for 5 min, followed by a 15 min testing session. The sound level was calibrated against an external dB meter and the output from the piezoelectric platform was checked in millivolts by oscilloscope. Three trial types were randomly presented: 1) 118 dB 40 ms pulse, 2) 4, 8 or 12 dB above background 5 ms prepulse (actual 74, 77 and 82 dB) followed by a 118 dB 40 ms pulse stimulus separated by 100 ms in each case, 3) no stimulus. Twenty-four presentations of the 118 dB pulse trial type, 10 presentations of each prepulse trial type, and 54 presentations of the no stimulus trial type occurred during each animal testing session in a constant order with an average inter-trial interval of 15 s. Lighting conditions within the accelerometer box were controlled for each cycle, with all testing occurring in a light chamber since all mice were tested 2–3 hours into the light cycle. Animal enclosures within the test cabinet were an appropriate size to prevent restraint stress during startle sessions. The data collected included the highest voltage during the response window (Vmax) and PPI, the amount of response after trials that contained the prepulse signal. The median of both measures was determined for each trial type in each animal and then averaged within a treatment group. PPI was computed within each animal as the percentage difference in voltage response in the prepulse condition divided by the voltage response in the non-prepulse condition. The latency for this maximal response was referred to as Tmax and may be viewed as an index of nerve conduction velocity (Desbrosses et al., 2006).

### Statistics

Averages from the electrophysiology preparations were obtained using the three largest intensities from each slice. Mean population spike amplitudes were subjected to repeated measures ANOVA with the factors Group (i.e., Thy1 KO mice and Control) and drug (before and during treatment). Significance levels were set at *p* < 0.05 for all *post-hoc* comparisons.

Data from the ASR and PPI were examined for normality of distribution. The Vmax, PPI and Tmax data were generally not normally distributed within an animal and testing session, so the median of the 10 replicates within an animal testing session was used as a value to represent each animal’s values. Group values were log-transformed for statistical comparisons and then antilogs recomputed for data expressed in graphs as means ± SEM. Data were analyzed with *t*-tests for two-group comparisons and with repeated measures ANOVA for comparison of groups with repeated measures. Significance levels were set at *p* < 0.05 for all *post-hoc* comparisons.

## Results

### Corticostriatal Population Spikes

**Figure 2.**
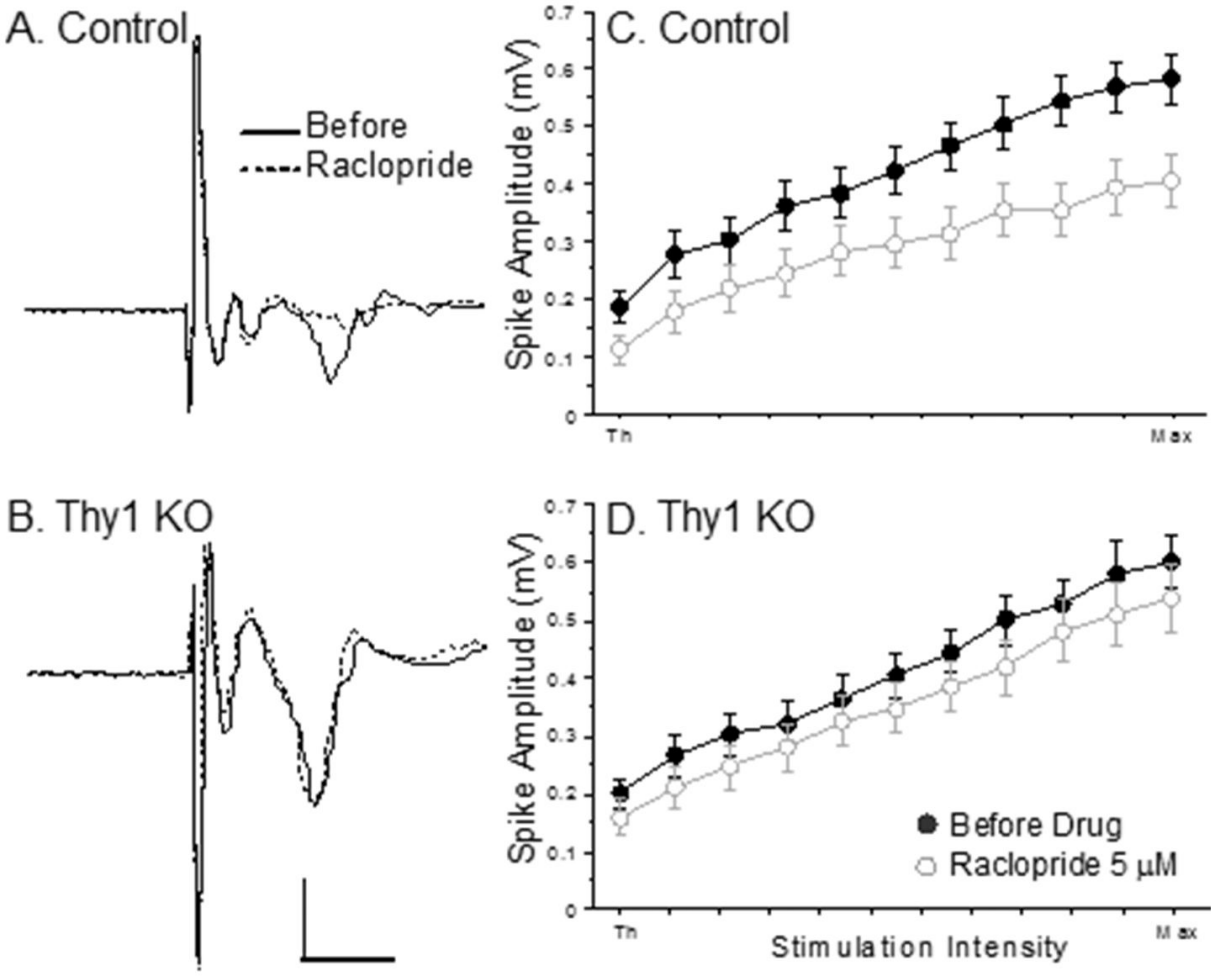
The D_2_ antagonist raclopride (5 μM) reduced synaptic responses in the control slices but not in the Thy1 KO slices. A and B Population spikes from exemplar control and Thy1 KO slices before and during perfusion of 5 μM raclopride in the bath. C and D Mean IO curves for Control and Thy1 KO mice slices before and during perfusion with raclopride. Bars show S.E.M (Repeated measures ANOVA with the factors Group and Treatment [*F*(1, 48) = 4.547, *p* = 0.0381]).

Thy1 KO (n = 25) and Control (n = 25) slices were subjected to the D_2_ receptor antagonist raclopride (5 μM). Raclopride reduced the population spike amplitude of the control slices much more than the Thy1 KO slices. Figures 2A and B show representative examples of population spikes before and during treatment with raclopride. Panels C and D show that raclopride reduced the IO curve of the control slices, but had little effect on the Thy1 KO slices. Averages were obtained using the three largest intensities from each slice. These mean population spike amplitudes were subjected to repeated measures ANOVA with the factors Group (i.e., Thy1 KO and Control) and Treatment [*F*(1, 48) = 4.547, *p* = 0.0381]. The post hoc analysis revealed that raclopride reduced the population spike amplitude of the controls but not the Thy1 KO slices.

**Figure 3.**
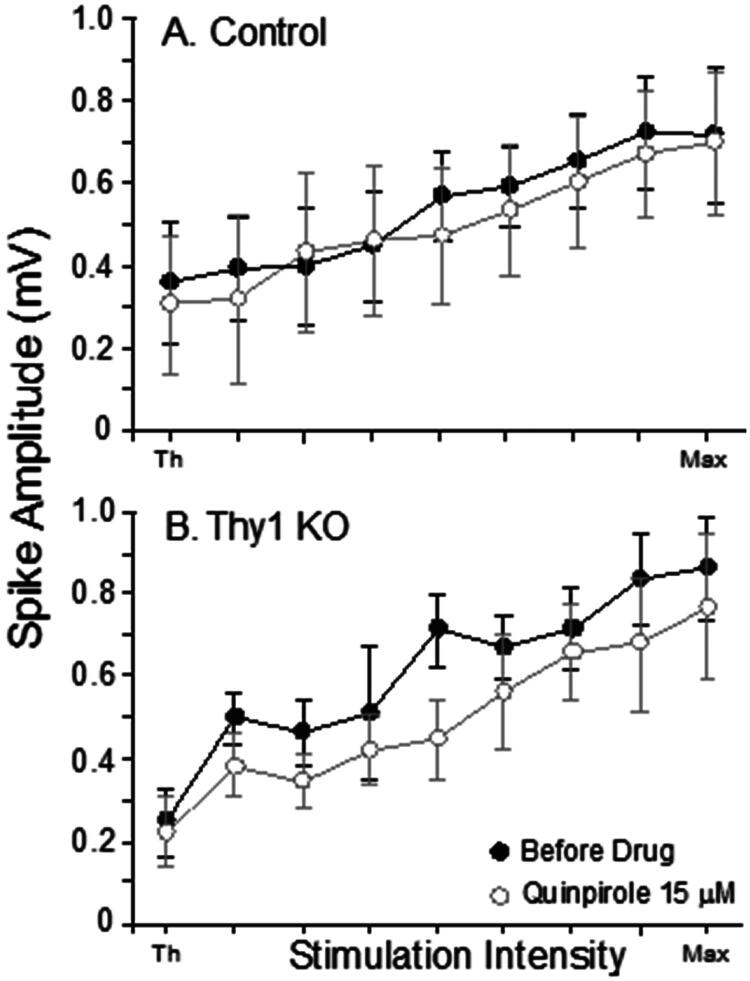
The D_2_ agonist quinpirole did not alter synaptic responses in Control or Thy1 KO slices. A and B: Mean IO curves for all Thy1 KO and Control slices during perfusion with 15 μM quinpirole. Lower doses of quinpirole (0.5 and 3 μM) also produced no alterations in synaptic transmission (data not shown). Bars show S.E.M.

Dopaminergic receptor activity was further examined using the D_2_ agonist quinpirole. Corticostriatal slices were subjected to 0.5 μM (Thy1 KO, n = 23; Control, n = 22), 3 μM (Thy1 KO, n = 16; Control, n = 17), or 15 μM quinpirole (Thy1 KO, n = 7; Control, n = 7). Similar to the previous analysis, the means of the maximum population spike amplitude were subjected to repeated measures ANOVA for each of the three concentrations of quinpirole. These analyses revealed no significant effects or interactions at any concentration of quinpirole. The results are shown in Figures 3A and 3B.

**Figure 4.**
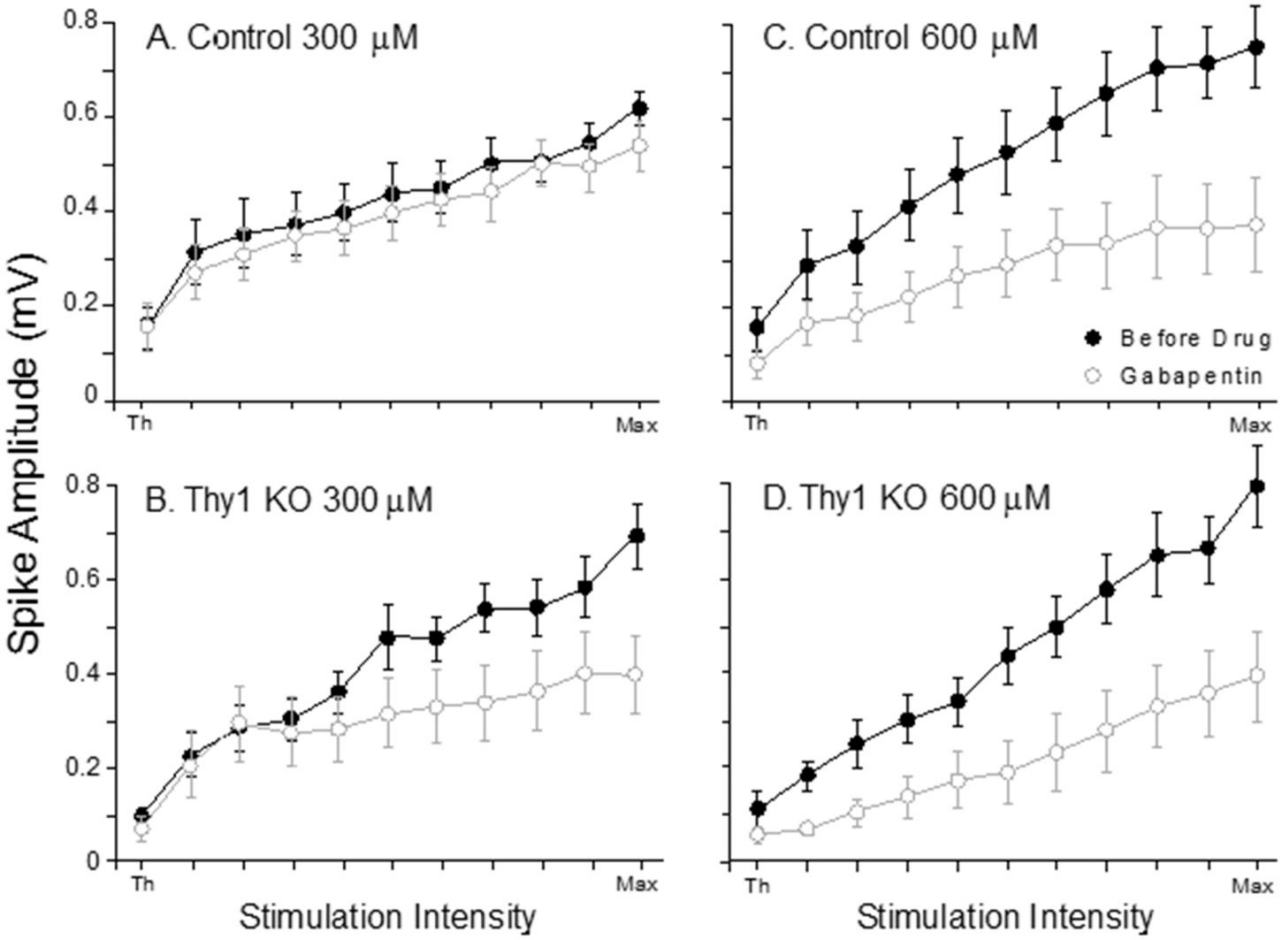
A low dose of gabapentin reduced synaptic responses in Thy1 KO, but did not affect control responses. Mean IO curves for Thy1 KO and Control slices perfused with 300 μM (A and B, Post hoc analysis [*F*(1, 18) = 4.635, *p* = 0.0451]) or 600 μM (C and D, Post hoc analysis [*F*(1, 18) = 23.114, *p* = 0.0002]) gabapentin. Bars show S.E.M.

Thy1 KO slices were also examined in the presence of gabapentin using a 300 μM dose (Thy1 KO, n = 10; Control, n = 10), and a 600 μM dose (Thy1 KO, n = 9; Control, n = 9). The 300 μM dose of gabapentin reduced the amplitude of the population spikes in Thy1 KO but did not affect the control slices. At a dose of 600 μM, gabapentin reduced the amplitude of the population spikes in both groups (Figure 4). The mean maximum population spike amplitudes were subjected to repeated measures ANOVAs for each concentration of gabapentin. A significant interaction of treatment and genotype was found for the 300 µM dose of gabapentin. The post hoc analysis revealed that the 300 μM gabapentin dose reduced the population spike amplitude in the Thy1 KO slices [*F*(1, 18) = 4.635, *p* = 0.0451] but not in the controls. Analysis of the 600 μM data revealed a significant effect of treatment, [*F*(1, 18) = 23.114, *p* = 0.0002], but the effect was not specific to mouse type.

### Startle Behavior

**Figure 5.**
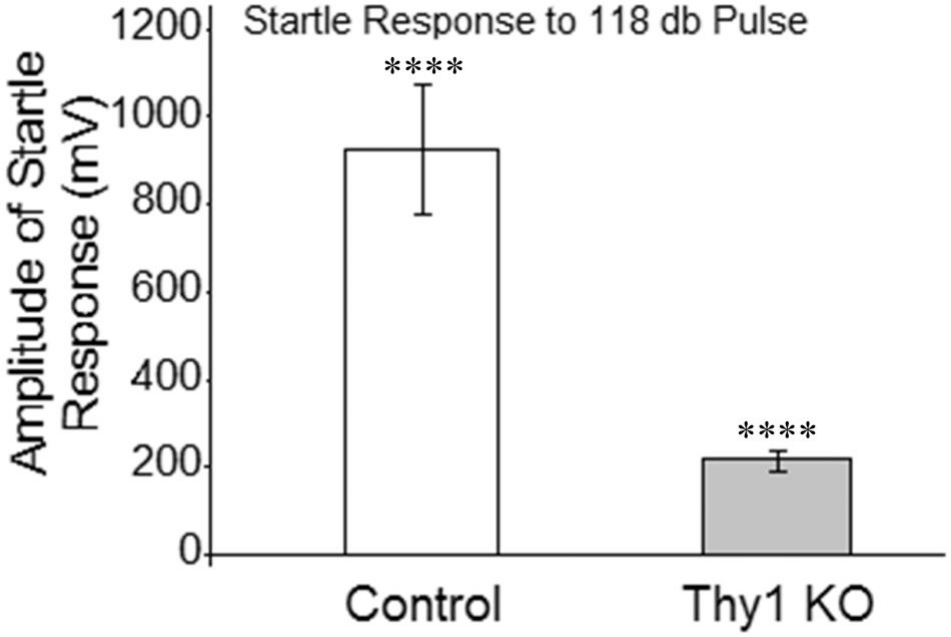
Startle response (Vmax; mean ± SEM mV) to 118 dB pulse in Thy1 KO and C57BL/6 male mice. Thy1 KO mice showed a significant reduction in startle response to the acoustic signal compared to control mice.

To attempt to associate the changes in the electrophysiological patterns with a behavioral phenotype, we used ASR (Vmax) and PPI to examine striatum-dependent sensorimotor activity in Thy1 KO (n = 11) and control (n = 8) mice. Thy1 KO mice showed a dramatic reduction in ASR compared to wild-type mice (Figure 5). The maximum amplitude of response to the acoustic startle was 215.8 ± 24.5 in Thy1 KO mice and 921.0 ± 140.29 in controls. This reduction in the ASR demonstrates that the Thy1 KO mice have a significant increase in inhibition of the basic startle reflex [t = 5.072; *p* = 0.0001].

**Figure 6.**
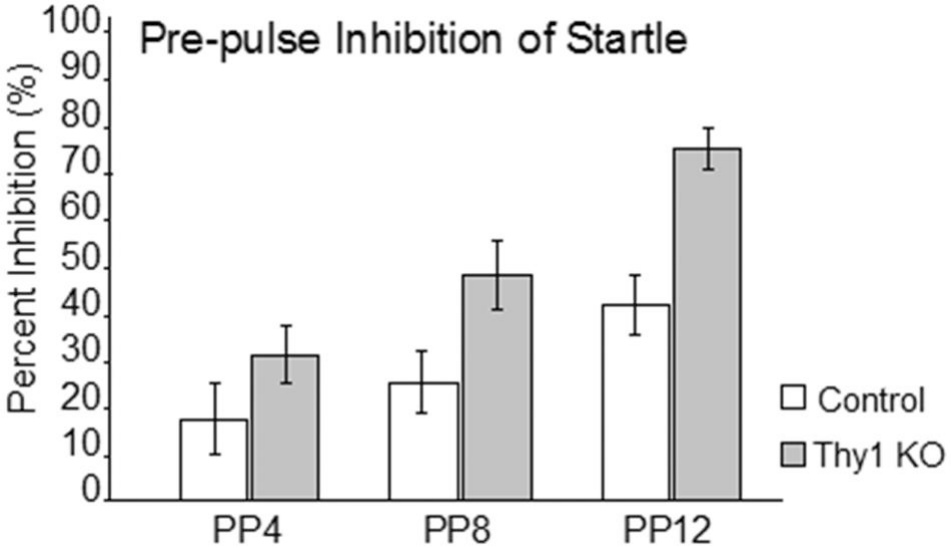
Percentage inhibition of acoustic startle response with pre-pulse signals of 4, 8, and 12 dB above threshold (mean ± SEM%). Thy1 KO mice had a significantly greater inhibition of startle responses than controls with all three prepulse tones (Between group analyses [F(1, 17) = 11.45, *p* = 0.035]; repeated measure analyses [F(2, 17) = 33.99, *p* = 0.0001]).

Prepulse acoustic signals diminished the magnitude of the ASR between 30 and 75% in Thy1 KO mice as the magnitude of the prepulse tone increased from 4 to 12 dB. In contrast, wild-type control mice only showed inhibition that ranged from 16 to 41% and significantly smaller amounts of inhibition compared to Thy1 KO mice (Figure 6). Between-group analysis [F(1, 17) = 11.45, *p* = 0.035] and especially, category for repeated measurements [F(2, 17) = 33.99, *p* = 0.0001] showed statistically significant differences, whereas the interaction between group and repeated-measures category did not reach statistical significance [F(2, 17) = 2.82, *p* = 0.073]. This greater inhibition of startle in Thy1 KO mice is consistent with the dramatically attenuated ASR.

Thy1 KO mice did not exhibit a statistically significant difference in the latency to the maximal startle response. The mean Latency of startle response (Tmax) in Thy1 KO mice was 15.39 ± 0.8 ms, while that of wild-type mice was 12.76 ± 1 ms [t = 2.028; *p* = 0.58].

## Discussion

The main objective of this study was to evaluate the loss of Thy1 on the striatal dopamine functions and the effect of gabapentin to provide some insights into the loss of this important synaptic protein in the brain. For this reason, we investigated the effect of the Thy1 loss on the striatal network-level synaptic activity and startle behavior accepted as modulated from the striatum. The specific choice to analyze dopaminergic agents and gabapentin was driven by our interest in how an inability of Thy1 expression influences dopaminergic and GABAergic functions, and their balance, which is critical for proper striatal function. The electrophysiological data show that the absence of Thy1 is associated with reduced sensitivity to D_2_-receptor antagonists as well as increased sensitivity to gabapentin. Thus, these data indicate that there is reduced D_2_ inhibition in cortico-striatal pathways in the absence of Thy1 and imply VDCCs, which may be affected. Behaviorally, the loss of Thy1 was associated with decreased startle responses and increased pre-pulse inhibition compared to controls. Our data are consistent with previous studies that proposed a role of Thy1 in the nigrostriatal dopaminergic modulation (Connor et al., 2008; Morris, 1985; Shults & Kimber, 1993) and expand the findings to suggest a functional relationship between Thy1 and VDCC (Alvarez-Laviada et al., 2014; Davies et al., 2010; Gravielle, 2021) as well as Thy1 and striatal-dependent behavior (Kasahara et al., 2015; Wang et al., 2014).

### Electrophysiology

The present electrophysiological results showed that raclopride application decreased the amplitude of spikes in wild-type animals, but did not affect it in Thy1 KO mice at the doses investigated. These results suggest that there may be a deficit in D_2_-dependent efficacy in the corticostriatal pathways in the absence of Thy1. Previous work from our laboratory demonstrated that Thy1 KO mice exhibit increased striatal expression of D_2_ receptors and the dopamine transporter (DAT), accompanied by impaired dopaminergic synaptic handling, including reduced [³H]dopamine loading and diminished dopamine release from synaptosomes (Connor et al., 2008). This pattern was interpreted as a compensatory upregulation of D_2_ receptors and DAT in the context of disrupted dopaminergic signaling, rather than a gain of D_2_ receptor function. Importantly, this biochemical profile provides a mechanistic framework that supports our present electrophysiological findings. Specifically, tonic D_2_-mediated modulation by endogenous dopamine is preserved in wild-type slices—allowing raclopride to reduce population spike amplitude—but is weakened in Thy1 KO slices, resulting in a blunted raclopride effect. Clinically, treatment with D_2_ agonist ropinirole effectively alters the disinhibition of the corticostriatal terminals in RLS patients (Trenkwalder, 2006; Winkelman, 2006). In our study, the D_2_ selective agonist quinpirole did not affect synaptic responses either in Thy1 KO nor in control animals. Previous studies have shown a similar lack of effect of the D_2_ agonists in the corticostriatal circuits in normal mice unless GABAergic tone or other network parameters are experimentally manipulated (Bamford et al., 2004; Garcia-Munoz et al., 1991a, 1991b). Our data suggest that the decreased expression of Thy1 in the RLS patients may not influence their response to the D_2_ agonists. However, we cannot rule out that our findings are influenced by a system where dopamine release and downstream signaling are compromised, as observed in Thy1 KO mice, exogenous D_2_ agonists may have limited efficacy despite apparent receptor upregulation (Connor et al., 2008).

Electrophysiological data on gabapentin-treated slices are consistent with findings pointing to the importance of GPI anchoring proteins for the function of VDCCs (Alvarez-Laviada et al., 2014; Davies et al., 2010). Gabapentin has a positive influence on the resistant cases of RLS (Winkelman, 2006). Gabapentin binds to the α2δ subunits 1 and 2 of the VDCCs reducing Ca^2+^ influx and activating K^+^ currents into the nerve terminals, which effectively decreases signaling for neurotransmitter release (Bertrand et al., 2003; Chen et al., 2018; Risher & Eroglu, 2020). Thy1 KO mice are hypersensitive to gabapentin compared to controls, as low doses of this drug decrease the amplitude of population spikes selectively in Thy1 KO slices.

Inappropriate interaction with VDCCs and altered sensitivity to dopaminergic (D_2_) input in the striatum in Thy1 KO mice, together with a shift toward increased inhibitory network tone (Mallet et al., 2019) could be the critical reason for hypersensitivity of Thy1 KO mice striatal networks to gabapentin. Additional mechanisms, such as a subsequent hyperactivation of the corticostriatal glutamatergic downstream, should also be at play that can be critical for a stronger gabapentin effect in Thy1 KO mice (Bamford et al., 2004; Gardoni & Bellone, 2015). Furthermore, it should be noted that both of these, glutamatergic and GABAergic neurotransmission, can be directly affected by VDCCs, through regulation of presynaptic Ca^2+^ influx and transmitter release (Chen et al., 2018; Gravielle, 2021; Rogawski & Taylor, 2006). Although early studies suggested that gabapentin might affect GABA metabolism (Goldlust et al., 1995), subsequent work has established that its primary mechanism of action is mediated by binding to the α2δ subunit of VDCCs, leading predominantly to reduced excitatory drive rather than direct stimulation of GABA synthesis (Bertrand et al., 2003; Chen et al., 2018; Risher & Eroglu, 2020). In this context, gabapentin is likely to exert stronger functional effects in circuits where inhibitory tone is already dominant, through secondary, network-level modulation of inhibitory–excitatory balance (Connor et al., 2008; Mallet et al., 2019). This framework may help to explain why RLS patients, who are also reported to have altered dopaminergic function in the striatum, positively respond to gabapentin treatment (Connor et al., 2008).

### Startle Behavior

The ASR is a subconscious motor response to unexpected noise that involves many brain regions responsible for auditory perception, motor action, and emotion (Davies et al., 2010; Lauer et al., 2017) and its evaluation can provide important information about the functional condition of certain structures. PPI of the startle response is used as a measuring tool for sensorimotor gating (Schilke et al., 2022; Winslow et al., 2002). The startle behavior was previously found to express dependence on the striatal dysfunctions and/or dopamine profile alterations of the subcortical structures (Davis et al., 2008; Napolitano et al., 2018). In our research, the startle behavior was significantly altered in the Thy1 KO mice compared to controls. Particularly, the Thy1 KO mice had less ASR and greater PPI, indicating the motor dysfunctions and abnormally greater sensorimotor inhibition associated with Thy1 loss and consequent functional alterations in the striatum.

Although Thy1 KO mice are not intended as a behavioral model of RLS, findings that showed decreased Thy1 expression in the brain of ID rats and in the brain of postmortem RLS patients (Batra & Seth, 2002; Wang et al., 2004), as well as changes in the dopaminergic profile in the striatum of Thy1 KO mice and RLS (Allen & Earley, 2007; Connor et al., 2008; Earley et al., 2014) indicated at least several common mechanisms for the consequent behavioral dysfunctions associated with Thy1 loss or lack in those models and RLS patients.

Striatal alterations in ID mice result in an increase in the frequency of ASR and shorter latency for their generation (Frauscher et al., 2007). Some studies of nutritional ID in rats showed decrease in both ASR (Burhans et al., 2006; Unger et al., 2006) and PPI (Pisansky et al., 2013), corresponding to the later findings indicating no linear relationship between ASR and PPI (Shoji & Miyakawa, 2018). In RLS patients mean ASR was significantly larger, generated more frequently and the latency was shorter compared to controls (Frauscher et al., 2007). The other work, where eyeblink reflexes were measured in RLS patients, showed destructed differential responding to the auditory stimuli, that requires proper responsiveness to one type of stimuli when inhibiting responses to others (McEchron et al., 2010). However, no impairment of auditory or tactile PPI was detected in patients with RLS (Leon-Sarmiento et al., 2015). Within this context, our behavioral findings indicate that loss of Thy1 alone is insufficient to reproduce the full behavioral phenotype of RLS. Nevertheless, the observed alterations in ASR and PPI provide important insight into the contribution of Thy1 to striatal inhibitory tone and the regulation of sensorimotor gating.

For interpreting the independent profile of the striatum in Thy1 KO mice in the context of startle behavior, it would be helpful to observe the dopaminergic and GABAergic impacts first; however, naturally, they affect each other and their effects are usually shared (Roberts et al., 2021; Swerdlow et al., 1990). For example, it has been shown that disrupted PPI is associated with disrupted GABA inhibition at the striatal and/or pallidal level, which itself is mediated via presynaptic D_2_ receptors (Centonze et al., 2003; Wiltschko et al., 2010). Ropinirole, a dopaminergic agonist of D_2_, D_3_ and D_4_, reduces PPI of eyeblink startle response in healthy male persons (Giakoumaki et al., 2007), while haloperidol, a dopaminergic D_2_ antagonist, fails to increase PPI in subjects with low levels of PPI but attenuates it in subjects with high PPI (Csomor et al., 2008). Map2K5 mutant mice, which were previously associated with RLS disorder (Li et al., 2017) and showed decreased dopaminergic cell survival in the nigrostriatal pathway, displayed increased ASR, decreased exploratory activity and PPI, especially in male mice. However, some motor disruptions were denoted in females as well (Huang et al., 2021). Heterozygous Meis1-KO mice, another potential model for RLS, showed not only a deficit of PPI but also hyposensitivity to the PPI-reducing effect of the dopamine agonist (German Mouse Clinic Consortium, 2017). At some point, a similar pattern was also shown in A11 (diencephalic-spinal dopaminergic nucleus) injured animals, one more considered model for RLS studies, where increased locomotion in a novel environment has been shown, pointing to the disinhibited motor centers (Ondo et al., 2000; Qu et al., 2007).

Earlier biochemical analysis contributes to our behavioral data as increased expression of D_2_ receptors, a loss of D_2_ receptor-associated proteins and decreased release of dopamine by synaptosomes in the striatum of Thy1 KO mice (Connor et al., 2008) make the condition that promotes GABAergic neurotransmission inside the striatum and create appropriate pattern of behavior. This point accords with our electrophysiological findings as Thy1 KO slices show dopaminergic D_2_ dysfunction and hypersensitivity to gabapentin; Alternatively, opposite behavioral alterations in nutritional ID rats are mediated by decreased expression of the dopamine transporter and elevated dopamine that downregulate D_2_ receptors (Erikson et al., 2002; Ward et al., 1995) and decrease glutamate and GABA metabolism (Batra & Seth, 2002; Erikson et al., 2002; Taneja et al., 1986) in the striatum.

Many clinical and scientific studies have shown unequivocally that an active striatum is linked to a decline in motor responsiveness and vice versa (Regan et al., 2019; Vink et al., 2005; Zandbelt & Vink, 2010). The behavioral phenotype revealed in Thy1 KO mice that it corresponds to an activated striatum, which means that there is activated most of its structures (> 90%) that are GABAergic medium spiny neurons (MSN) (Hikosaka et al., 1989). They spread their influence in intrinsic lateral inhibitory pathways by feedback inhibition and by feedforward inhibition with other GABAergic interneurons (Orduz et al., 2013). Thus, the striatum in Thy1 KO mice has disinhibited GABAergic neurons, which by their increased activity, produce an appropriate behavioral phenotype.

## Conclusions

Finally, we can conclude that the absence of Thy1 in mice is associated with compromised dopaminergic modulation of the corticostriatal synaptic transmission and involves D_2_ receptors. On the other hand, hypersensitivity to gabapentin in Thy1 KO mice may point to disinhibited GABAergic neurotransmission, which, *per se*, causes significant alterations of the striatal circuitry. As a result, behaviorally, Thy1 KO mice have decreased startle reactions and increased PPI compared to controls, which corresponds to the disrupted dopaminergic modulation of the corticostriatal downstream pathway and parallel intrinsic processes. Our data are consistent with the earlier findings demonstrating impacted dopaminergic functions in the striatum and other brain structures after loss of Thy1 (Connor et al., 2008; Morris, 1985; Shults & Kimber, 1993) and the role of dopaminergic dysfunction in the functions of the striatum. At the same time, our data indicate the role of Thy1 in the functioning of the striatal targets of gabapentin, the critical therapeutic agent used in the treatment of some neurological disorders (Davies et al., 2010; Trenkwalder et al., 2018). Moreover, we demonstrated that Thy1 deficiency leads to specific imbalances in striatal function, which may be relevant to pathologies such as restless legs syndrome (RLS) and may influence behavioral outcomes and responsiveness to therapeutic strategies.

## References

[CIT0001] Allen, R. P., & Earley, C. J. (2007). The role of iron in restless legs syndrome. *Movement Disorders: official Journal of the Movement Disorder Society*, *22*(Suppl 18), S440–S8. 10.1002/mds.21607 17566122

[CIT0002] Alvarez-Laviada, A., Kadurin, I., Senatore, A., Chiesa, R., & Dolphin, A. C. (2014). The inhibition of functional expression of calcium channels by prion protein demonstrates competition with α2δ for GPI-anchoring pathways. *The Biochemical Journal*, *458*(2), 365–374. 10.1042/BJ20131405 24329154 PMC3924758

[CIT0003] Bamford, N. S., Robinson, S., Palmiter, R. D., Joyce, J. A., Moore, C., & Meshul, C. K. (2004). Dopamine modulates release from corticostriatal terminals. *The Journal of Neuroscience: The Official Journal of the Society for Neuroscience*, *24*(43), 9541–9552. 10.1523/JNEUROSCI.2891-04.2004 15509741 PMC6730145

[CIT0004] Barrière, G., Cazalets, J. R., Bioulac, B., Tison, F., & Ghorayeb, I. (2005). The restless legs syndrome. *Progress in Neurobiology*, *77*(3), 139–165. 10.1016/j.pneurobio.2005.10.007 16300874

[CIT0005] Batra, J., & Seth, P. K. (2002). Effect of iron deficiency on developing rat brain. *Indian Journal of Clinical Biochemistry: IJCB*, *17*(2), 108–114. 10.1007/BF02867982 23105361 PMC3454122

[CIT0006] Bertrand, S., Morin, F., & Lacaille, J. C. (2003). Different actions of gabapentin and baclofen in hippocampus from weaver mice. *Hippocampus*, *13*(4), 525–528. 10.1002/hipo.10131 12836919

[CIT0007] Breton, A. B., Fox, J. A., Brownson, M. P., & McEchron, M. D. (2015). Postnatal nutritional iron deficiency impairs dopaminergic-mediated synaptic plasticity in the CA1 area of the hippocampus. *Nutritional Neuroscience*, *18*(6), 241–247. 10.1179/1476830514Y.0000000121 24678581

[CIT0008] Burhans, M. S., Dailey, C., Wiesinger, J., Murray-Kolb, L. E., Jones, B. C., & Beard, J. L. (2006). Iron deficiency affects acoustic startle response and latency, but not prepulse inhibition in young adult rats. *Physiology & Behavior*, *87*(5), 917–924. 10.1016/j.physbeh.2006.02.014 16603209

[CIT0009] Calabresi, P., Pisani, A., Centonze, D., & Bernardi, G. (1997). Synaptic plasticity and physiological interactions between dopamine and glutamate in the striatum. *Neuroscience and Biobehavioral Reviews*, *21*(4), 519–523. 10.1016/s0149-7634(96)00029-2 9195611

[CIT0010] Centonze, D., Grande, C., Usiello, A., Gubellini, P., Erbs, E., Martin, A. B., Pisani, A., Tognazzi, N., Bernardi, G., Moratalla, R., Borrelli, E., & Calabresi, P. (2003). Receptor subtypes involved in the presynaptic and postsynaptic actions of dopamine on striatal interneurons. *The Journal of Neuroscience: The Official Journal of the Society for Neuroscience*, *23*(15), 6245–6254. 10.1523/JNEUROSCI.23-15-06245.2003 12867509 PMC6740558

[CIT0011] Chen, J., Li, L., Chen, S. R., Chen, H., Xie, J. D., Sirrieh, R. E., MacLean, D. M., Zhang, Y., Zhou, M. H., Jayaraman, V., & Pan, H. L. (2018). The α2δ-1-NMDA Receptor Complex Is Critically Involved in Neuropathic Pain Development and Gabapentin Therapeutic Actions. *Cell Reports*, *22*(9), 2307–2321. 10.1016/j.celrep.2018.02.021 29490268 PMC5873963

[CIT0012] Cheng, J. K., & Chiou, L. C. (2006). Mechanisms of the antinociceptive action of gabapentin. *Journal of Pharmacological Sciences*, *100*(5), 471–486. 10.1254/jphs.cr0050020 16474201

[CIT0013] Connor, J. R., Wang, X. S., Allen, R. P., Beard, J. L., Wiesinger, J. A., Felt, B. T., & Earley, C. J. (2009). Altered dopaminergic profile in the putamen and substantia nigra in restless leg syndrome. *Brain: a Journal of Neurology*, *132*(Pt 9), 2403–2412. 10.1093/brain/awp125 19467991 PMC2732265

[CIT0014] Connor, J. R., Wang, X. S., Neely, E. B., Ponnuru, P., Morita, H., & Beard, J. (2008). Comparative study of the influence of Thy1 deficiency and dietary iron deficiency on dopaminergic profiles in the mouse striatum. *Journal of Neuroscience Research*, *86*(14), 3194–3202. 10.1002/jnr.21758 18615641

[CIT0015] Csomor, P. A., Stadler, R. R., Feldon, J., Yee, B. K., Geyer, M. A., & Vollenweider, F. X. (2008). Haloperidol differentially modulates prepulse inhibition and p50 suppression in healthy humans stratified for low and high gating levels. *Neuropsychopharmacology: official Publication of the American College of Neuropsychopharmacology*, *33*(3), 497–512. 10.1038/sj.npp.1301421 17460616

[CIT0016] Davies, A., Kadurin, I., Alvarez-Laviada, A., Douglas, L., Nieto-Rostro, M., Bauer, C. S., Pratt, W. S., & Dolphin, A. C. (2010). The alpha2delta subunits of voltage-gated calcium channels form GPI-anchored proteins, a posttranslational modification essential for function. *Proceedings of the National Academy of Sciences of the United States of America*, *107*(4), 1654–1659. 10.1073/pnas.0908735107 20080692 PMC2824380

[CIT0017] Davis, M., Antoniadis, E. A., Amaral, D. G., & Winslow, J. T. (2008). Acoustic startle reflex in rhesus monkeys: a review. *Reviews in the Neurosciences*, *19*(2–3), 171–185. 10.1515/revneuro.2008.19.2-3.171 18751523

[CIT0018] De Haan, M. I. C., van Well, S., Visser, R. M., Scholte, H. S., van Wingen, G. A., & Kindt, M. (2018). The influence of acoustic startle probes on fear learning in humans. *Scientific Reports*, *8*(1), 14552. 10.1038/s41598-018-32646-1 30267018 PMC6162305

[CIT0019] Desbrosses, K., Babault, N., Scaglioni, G., Meyer, J. P., & Pousson, M. (2006). Neural activation after maximal isometric contractions at different muscle lengths. *Medicine and Science in Sports and Exercise*, *38*(5), 937–944. 10.1249/01.mss.0000218136.58899.46 16672848

[CIT0020] Earley, C. J., Connor, J., Garcia-Borreguero, D., Jenner, P., Winkelman, J., Zee, P. C., & Allen, R. (2014). Altered brain iron homeostasis and dopaminergic function in Restless Legs Syndrome (Willis-Ekbom Disease). *Sleep Medicine*, *15*(11), 1288–1301. 10.1016/j.sleep.2014.05.009 25201131

[CIT0021] Earley, C. J., Kuwabara, H., Wong, D. F., Gamaldo, C., Salas, R. E., Brašić, J. R., Ravert, H. T., Dannals, R. F., & Allen, R. P. (2013). Increased synaptic dopamine in the putamen in restless legs syndrome. *Sleep*, *36*(1), 51–57. 10.5665/sleep.2300 23288971 PMC3524542

[CIT0022] Erikson, K. M., Shihabi, Z. K., Aschner, J. L., & Aschner, M. (2002). Manganese accumulates in iron-deficient rat brain regions in a heterogeneous fashion and is associated with neurochemical alterations. *Biological Trace Element Research*, *87*(1–3), 143–156. 10.1385/BTER:87:1-3:143 12117224

[CIT0023] Frauscher, B., Löscher, W., Högl, B., Poewe, W., & Kofler, M. (2007). Auditory startle reaction is disinhibited in idiopathic restless legs syndrome. *Sleep*, *30*(4), 489–493. 10.1093/sleep/30.4.489 17520793

[CIT0024] Garcia-Munoz, M., Young, S. J., & Groves, P. M. (1991a). Terminal excitability of the corticostriatal pathway. I. Regulation by dopamine receptor stimulation. *Brain Research*, *551*(1-2), 195–206. 10.1016/0006-8993(91)90933-m 1913151

[CIT0025] Garcia-Munoz, M., Young, S. J., & Groves, P. M. (1991b). Terminal excitability of the corticostriatal pathway. II. Regulation by glutamate receptor stimulation. *Brain Research*, *551*(1-2), 207–215. 10.1016/0006-8993(91)90934-n 1680522

[CIT0026] Gardoni, F., & Bellone, C. (2015). Modulation of the glutamatergic transmission by Dopamine: a focus on Parkinson, Huntington and Addiction diseases. *Frontiers in Cellular Neuroscience*, *9*, 25. 10.3389/fncel.2015.00025 25784855 PMC4345909

[CIT0027] Giakoumaki, S. G., Roussos, P., Frangou, S., & Bitsios, P. (2007). Disruption of prepulse inhibition of the startle reflex by the preferential D(3) agonist ropinirole in healthy males. *Psychopharmacology*, *194*(3), 289–295. 10.1007/s00213-007-0843-7 17579840

[CIT0028] Goldlust, A., Su, T. Z., Welty, D. F., Taylor, C. P., & Oxender, D. L. (1995). Effects of anticonvulsant drug gabapentin on the enzymes in metabolic pathways of glutamate and GABA. *Epilepsy Research*, *22*(1), 1–11. 10.1016/0920-1211(95)00028-9 8565962

[CIT0029] Gravielle, M. C. (2021). Regulation of GABAA receptors induced by the activation of L-type voltage-gated calcium channels. *Membranes*, *11*(7), 486. 10.3390/membranes11070486 34209589 PMC8304739

[CIT0030] Happe, S., Sauter, C., Klösch, G., Saletu, B., & Zeitlhofer, J. (2003). Gabapentin versus ropinirole in the treatment of idiopathic restless Legs Syndrome. *Neuropsychobiology*, *48*(2), 82–86. 10.1159/000072882 14504416

[CIT0031] Hawes, S. L., Gillani, F., Evans, R. C., Benkert, E. A., & Blackwell, K. T. (2013). Sensitivity to theta-burst timing permits LTP in dorsal striatal adult brain slice. *Journal of Neurophysiology*, *110*(9), 2027–2036. 10.1152/jn.00115.2013 23926032 PMC4073966

[CIT0032] Hikosaka, O., Sakamoto, M., & Usui, S. (1989). Functional properties of monkey caudate neurons. III. Activities related to expectation of target and reward. *Journal of Neurophysiology*, *61*(4), 814–832. 10.1152/jn.1989.61.4.814 2723722

[CIT0033] Horne, A. L., Woodruff, G. N., & Kemp, J. A. (1990). Synaptic potentials mediated by excitatory amino acid receptors in the nucleus accumbens of the rat, *in vitro*. *Neuropharmacology*, *29*(10), 917–921. 10.1016/0028-3908(90)90142-e 1979429

[CIT0034] Huang, Y., Wang, P., Morales, R., Luo, Q., & Ma, J. (2021). Map2k5-deficient mice manifest phenotypes and pathological changes of dopamine deficiency in the central nervous system. *Frontiers in Aging Neuroscience*, *13*, 651638. 10.3389/fnagi.2021.651638 34168549 PMC8217467

[CIT0035] Hundehege, P., Fernandez-Orth, J., Römer, P., Ruck, T., Müntefering, T., Eichler, S., Cerina, M., Epping, L., Albrecht, S., Menke, A. F., Birkner, K., Göbel, K., Budde, T., Zipp, F., Wiendl, H., Gorji, A., Bittner, S., & Meuth, S. G. (2018). Targeting voltage-dependent calcium channels with pregabalin exerts a direct neuroprotective effect in an animal model of multiple sclerosis. *Neuro-Signals*, *26*(1), 77–93. 10.1159/000495425 30481775

[CIT0036] Kasahara, Y., Arime, Y., Hall, F. S., Uhl, G. R., & Sora, I. (2015). Region-specific dendritic spine loss of pyramidal neurons in dopamine transporter knockout mice. *Current Molecular Medicine*, *15*(3), 237–244. 10.2174/1566524015666150330143613 25817859

[CIT0037] Kollias, G., Spanopoulou, E., Grosveld, F., Ritter, M., Beech, J., & Morris, R. (1987). Differential regulation of a Thy-1 gene in transgenic mice. *Proceedings of the National Academy of Sciences of the United States of America*, *84*(6), 1492–1496. 10.1073/pnas.84.6.1492 2882505 PMC304460

[CIT0038] Lauer, A. M., Behrens, D., & Klump, G. (2017). Acoustic startle modification as a tool for evaluating auditory function of the mouse: Progress, pitfalls, and potential. *Neuroscience and Biobehavioral Reviews*, *77*, 194–208. 10.1016/j.neubiorev.2017.03.009 28327385 PMC5446932

[CIT0039] Leon-Sarmiento, F. E., Peckham, E., Leon-Ariza, D. S., Bara-Jimenez, W., & Hallett, M. (2015). Auditory and lower limb tactile prepulse inhibition in primary restless Legs Syndrome: Clues to Its pathophysiology. *Journal of Clinical Neurophysiology*, *32*(4), 369–374. 10.1097/WNP.0000000000000196 26241246 PMC7293355

[CIT0040] Li, G., Tang, H., Wang, C., Qi, X., Chen, J., Chen, S., & Ma, J. (2017). Association of BTBD9 and MAP2K5/SKOR1 With Restless Legs Syndrome in Chinese Population. *Sleep*, *40*(4). 10.1093/sleep/zsx028 28329290

[CIT0041] Mallet, N., Delgado, L., Chazalon, M., Miguelez, C., & Baufreton, J. (2019). Cellular and Synaptic Dysfunctions in Parkinson’s Disease: Stepping out of the Striatum. *Cells*, *8*(9), 1005. 10.3390/cells8091005 31470672 PMC6769933

[CIT0042] Mann, D. A., Doherty, P., & Walsh, F. S. (1989). Increased intracellular cyclic AMP differentially modulates nerve growth factor induction of three neuronal recognition molecules involved in neurite outgrowth. *Journal of Neurochemistry*, *53*(5), 1581–1588. 10.1111/j.1471-4159.1989.tb08555.x 2571678

[CIT0043] McEchron, M. D., Alexander, D. N., Smith, M. E., Hoffman, D. L., Podskalny, G. D., & Connor, J. R. (2010). Altered eyeblink reflex conditioning in restless legs syndrome patients. *Sleep Medicine*, *11*(3), 314–319. 10.1016/j.sleep.2009.06.010 20149726

[CIT0044] Mercuri, N., Bernardi, G., Calabresi, P., Cotugno, A., Levi, G., & Stanzione, P. (1985). Dopamine decreases cell excitability in rat striatal neurons by pre- and postsynaptic mechanisms. *Brain Research*, *358*(1-2), 110–121. 10.1016/0006-8993(85)90954-0 2866815

[CIT0045] Michaud, M., Soucy, J. P., Chabli, A., Lavigne, G., & Montplaisir, J. (2002). SPECT imaging of striatal pre- and postsynaptic dopaminergic status in restless legs syndrome with periodic leg movements in sleep. *Journal of Neurology*, *249*(2), 164–170. 10.1007/pl00007859 11985381

[CIT0046] Morris, R. (1985). Thy-1 in developing nervous tissue. *Developmental Neuroscience*, *7*(3), 133–160. 10.1159/000112283 2866949

[CIT0047] Morris, R. J., Barber, P. C., Beech, J., & Raisman, G. (1983). The distribution of Thy-1 antigen in the P.N.S. of the adult rat. *Journal of Neurocytology*, *12*(6), 1017–1039. 10.1007/BF01153348 6141229

[CIT0048] Napolitano, F., D’Angelo, L., de Girolamo, P., Avallone, L., de Lange, P., & Usiello, A. (2018). The thyroid hormone-target gene Rhes a novel crossroad for neurological and psychiatric disorders: New insights from animal models. *Neuroscience*, *384*, 419–428. 10.1016/j.neuroscience.2018.05.027 29857029

[CIT0049] Nosten-Bertrand, M., Errington, M. L., Murphy, K. P., Tokugawa, Y., Barboni, E., Kozlova, E., Michalovich, D., Morris, R. G., Silver, J., Stewart, C. L., Bliss, T. V., & Morris, R. J. (1996). Normal spatial learning despite regional inhibition of LTP in mice lacking Thy-1. *Nature*, *379*(6568), 826–829. 10.1038/379826a0 8587606

[CIT0050] Ondo, W. G., He, Y., Rajasekaran, S., & Le, W. D. (2000). Clinical correlates of 6-hydroxydopamine injections into A11 dopaminergic neurons in rats: a possible model for restless legs syndrome. *Movement Disorders*, *15*(1), 154–158. 10.1002/1531-8257(200001)15:1<154:aid-mds1025>3.0.co;2-q 10634257

[CIT0051] Orduz, D., Bischop, D. P., Schwaller, B., Schiffmann, S. N., & Gall, D. (2013). Parvalbumin tunes spike-timing and efferent short-term plasticity in striatal fast spiking interneurons. *The Journal of Physiology*, *591*(13), 3215–3232. 10.1113/jphysiol.2012.250795 23551945 PMC3717224

[CIT0052] Pisansky, M. T., Wickham, R. J., Su, J., Fretham, S., Yuan, L. L., Sun, M., Gewirtz, J. C., & Georgieff, M. K. (2013). Iron deficiency with or without anemia impairs prepulse inhibition of the startle reflex. *Hippocampus*, *23*(10), 952–962. 10.1002/hipo.22151 23733517 PMC3888485

[CIT0053] Qu, S., Le, W., Zhang, X., Xie, W., Zhang, A., & Ondo, W. G. (2007). Locomotion is increased in a11-lesioned mice with iron deprivation: a possible animal model for restless legs syndrome. *Journal of Neuropathology and Experimental Neurology*, *66*(5), 383–388. 10.1097/nen.0b013e3180517b5f 17483695

[CIT0054] Regan, S. L., Hufgard, J. R., Pitzer, E. M., Sugimoto, C., Hu, Y. C., Williams, M. T., & Vorhees, C. V. (2019). Knockout of latrophilin-3 in Sprague-Dawley rats causes hyperactivity, hyper-reactivity, under-response to amphetamine, and disrupted dopamine markers. *Neurobiology of Disease*, *130*, 104494. 10.1016/j.nbd.2019.104494 31176715 PMC6689430

[CIT0055] Rege, T. A., & Hagood, J. S. (2006a). Thy-1 as a regulator of cell–cell and cell–matrix interactions in axon regeneration, apoptosis, adhesion, migration, cancer, and fibrosis. *FASEB Journal*, *20*(8), 1045–1054. 10.1096/fj.05-5460rev 16770003

[CIT0056] Rege, T. A., & Hagood, J. S. (2006b). Thy-1, a versatile modulator of signaling affecting cellular adhesion, proliferation, survival, and cytokine/growth factor responses. *Biochimica et Biophysica Acta*, *1763*(10), 991–999. Review. 10.1016/j.bbamcr.2006.08.008 16996153 PMC1781924

[CIT0057] Risher, W. C., & Eroglu, C. (2020). Emerging roles for α2δ subunits in calcium channel function and synaptic connectivity. *Current Opinion in Neurobiology*, *63*, 162–169. 10.1016/j.conb.2020.04.007 32521436 PMC7483897

[CIT0058] Roberts, B. M., Lopes, E. F., & Cragg, S. J. (2021). Axonal modulation of striatal dopamine release by local Gamma-Aminobutyric Acid (GABA) signalling. *Cells*, *10*(3), 709. 10.3390/cells10030709 33806845 PMC8004767

[CIT0059] Rogawski, M. A., & Taylor, C. P. (2006). Calcium channel α2–δ subunit, a new antiepileptic drug target. *Epilepsy Res*, *69*(3), 183–272. 10.1016/j.eplepsyres.2006.03.014 16835945 PMC1574365

[CIT0060] Salminen, A. V., Garrett, L., Schormair, B., Rozman, J., Giesert, F., Niedermeier, K. M., Becker, L., Rathkolb, B., Rácz, I., Klingenspor, M., Klopstock, T., Wolf, E., Zimmer, A., Gailus-Durner, V., Torres, M., Fuchs, H., Hrabě de Angelis, M., Wurst, W., Hölter, S. M., & Winkelmann, J, German Mouse Clinic Consortium. (2017). Meis1: effects on motor phenotypes and the sensorimotor system in mice. *Disease Models & Mechanisms*, *10*(8), 981–991. 10.1242/dmm.030080 28645892 PMC5560065

[CIT0061] Schilke, E. D., Tremolizzo, L., Appollonio, I., & Ferrarese, C. (2022). Tics: neurological disorders determined by a deficit in sensorimotor gating processes. *Neurological Sciences*, *43*(10), 5839–5850. 10.1007/s10072-022-06235-0 35781754 PMC9474467

[CIT0062] Shoji, H., & Miyakawa, T. (2018). Relationships between the acoustic startle response and prepulse inhibition in C57BL/6J mice: a large-scale meta-analytic study. *Molecular Brain*, *11*(1), 42–42. 10.1186/s13041-018-0382-7 30001725 PMC6044095

[CIT0063] Shults, C. W., & Kimber, T. A. (1993). Thy-1 immunoreactivity distinguishes patches/striosomes from matrix in the early postnatal striatum of the rat. *Brain Research. Developmental Brain Research*, *75*(1), 136–140. 10.1016/0165-3806(93)90073-j 7900932

[CIT0064] Stohl, W., & Gonatas, N. K. (1977). Distribution of the thy-1 antigen in cellular and subcellular fractions of adult mouse brain. *The Journal of Immunology*, *119*(2), 422–427. 10.4049/jimmunol.119.2.422 69659

[CIT0065] Swerdlow, N. R., Braff, D. L., & Geyer, M. A. (1990). GABAergic projection from nucleus accumbens to ventral pallidum mediates dopamine-induced sensorimotor gating deficits of acoustic startle in rats. *Brain Research*, *532*(1-2), 146–150. 10.1016/0006-8993(90)91754-5 2282510

[CIT0066] Taneja, V., Mishra, K., & Agarwal, K. N. (1986). Effect of early iron deficiency in rat on the gamma-aminobutyric acid shunt in brain. *Journal of Neurochemistry*, *46*(6), 1670–1674. 10.1111/j.1471-4159.1986.tb08483.x 2871128

[CIT0067] Trenkwalder, C. (2006). The weight of evidence for ropinirole in restless legs syndrome. *European Journal of Neurology*, *13*(s3), 21–30. 10.1111/j.1468-1331.2006.01588 16930379

[CIT0068] Trenkwalder, C., Allen, R., Högl, B., Clemens, S., Patton, S., Schormair, B., & Winkelmann, J. (2018). Comorbidities, treatment, and pathophysiology in restless legs syndrome. *The Lancet. Neurology*, *17*(11), 994–1005. 10.1016/S1474-4422(18)30311-9 30244828

[CIT0069] Unger, E. L., Bianco, L. E., Burhans, M. S., Jones, B. C., & Beard, J. L. (2006). Acoustic startle response is disrupted in iron-deficient rats. *Pharmacology, Biochemistry, and Behavior*, *84*(2), 378–384. 10.1016/j.pbb.2006.06.003 16828857

[CIT0070] Vink, M., Kahn, R. S., Raemaekers, M., van den Heuvel, M., Boersma, M., & Ramsey, N. F. (2005). Function of striatum beyond inhibition and execution of motor responses. *Human Brain Mapping*, *25*(3), 336–344. 10.1002/hbm.20111 15852388 PMC6871687

[CIT0071] Wang, L., Rodriguiz, R. M., Wetsel, W. C., Sheng, H., Zhao, S., Liu, X., Paschen, W., & Yang, W. (2014). Neuron-specific Sumo1-3 knockdown in mice impairs episodic and fear memories. *Journal of Psychiatry & Neuroscience: JPN*, *39*(4), 259–266. 10.1503/jpn.130148 24690371 PMC4074237

[CIT0072] Wang, X., Wiesinger, J., Beard, J., Felt, B., Menzies, S., Earley, C., Allen, R., & Connor, J. (2004). Thy1 expression in the brain is affected by iron and is decreased in Restless Legs syndrome. *Journal of the Neurological Sciences*, *220*(1-2), 59–66. 10.1016/j.jns.2004.02.004 15140607

[CIT0073] Ward, R. J., Dexter, D., Florence, A., Aouad, F., Hider, R., Jenner, P., & Crichton, R. R. (1995). Brain iron in the ferrocene-loaded rat: its chelation and influence on dopamine metabolism. *Biochemical Pharmacology*, *49*(12), 1821–1826. 10.1016/0006-2952(94)00521-m 7598744

[CIT0074] Wiltschko, A. B., Pettibone, J. R., & Berke, J. D. (2010). Opposite effects of stimulant and antipsychotic drugs on striatal fast-spiking interneurons. *Neuropsychopharmacology: official Publication of the American College of Neuropsychopharmacology*, *35*(6), 1261–1270. 10.1038/npp.2009.226 20090670 PMC3055348

[CIT0075] Winkelman, J. W. (2006). Considering the causes of RLS. *European Journal of Neurology*, *13*(s3), 8–14. Review. 10.1111/j.1468-1331.2006.01586 16930377

[CIT0076] Winslow, J. T., Parr, L. A., & Davis, M. (2002). Acoustic startle, prepulse inhibition, and fear-potentiated startle measured in rhesus monkeys. *Biological Psychiatry*, *51*(11), 859–866. 10.1016/s0006-3223(02)01345-8 12022958

[CIT0077] Zandbelt, B. B., & Vink, M. (2010). On the role of the striatum in response inhibition. *PloS One*, *5*(11), e13848. 10.1371/journal.pone.0013848 21079814 PMC2973972

